# ATF4 transcriptionally activates NUPR1 to promote ferroptosis in chondrocytes and osteoarthritis development

**DOI:** 10.1016/j.jphyss.2025.100039

**Published:** 2025-08-05

**Authors:** Chen Kuang, Taiyang Liao, Lishi Jie, Yibao Wei, Deren Liu, Enrui Hu, Liang Ding, Peimin Wang

**Affiliations:** a Department of Orthopedics and Traumatology, Affiliated Hospital of Nanjing University of Chinese Medicine, Jiangsu Provincial Hospital of Traditional Chinese Medicine, Nanjing, 210029 Jiangsu Province, PR China; b Key Laboratory for Metabolic Diseases in Chinese Medicine, Nanjing University of Chinese Medicine, Nanjing, 210023 Jiangsu Province, PR China; c Department of Orthopedics and Traumatology, Kunshan Affiliated Hospital of Nanjing University of Chinese Medicine, Kunshan, 215300 Jiangsu Province, PR China; d Department of Orthopedics and Traumatology, The Second Affiliated Hospital of Nanjing University of Chinese Medicine, Nanjing, 210017 Jiangsu, PR China

**Keywords:** Osteoarthritis, ATF4, Ferroptosis, NUPR1, Chondrocytes

## Abstract

Osteoarthritis (OA) is a common degenerative joint disease characterized by cartilage destruction and inflammation. This study reveals that activating transcription factor 4 (ATF4) is upregulated in IL-1β-treated chondrocytes and promotes ferroptosis, a form of programmed cell death. Knockdown of ATF4 alleviated cartilage damage and reduced ferroptosis in both cell and mouse models. Mechanistically, ATF4 directly binds to the promoter of nuclear protein 1 (NUPR1) and activates its transcription. Overexpression of NUPR1 reversed the protective effects of ATF4 knockdown, confirming the critical role of the ATF4-NUPR1 axis in mediating ferroptosis and OA progression. These findings identify ATF4 as a key driver of OA via ferroptosis regulation and suggest that targeting the ATF4-NUPR1 pathway may offer a promising therapeutic strategy.

## Highlights

• ATF4 is upregulated in IL-1β-treated chondrocytes and facilitates ferroptosis.
• Knockdown of ATF4 improves osteoarthritis by inhibiting ferroptosis in the DMM-induced osteoarthritis model.
• ATF4 transcriptionally activates NUPR1, offering a novel therapeutic strategy to mitigate ferroptosis and slow osteoarthritis progression.

## Graphical Abstract

**Figure:**
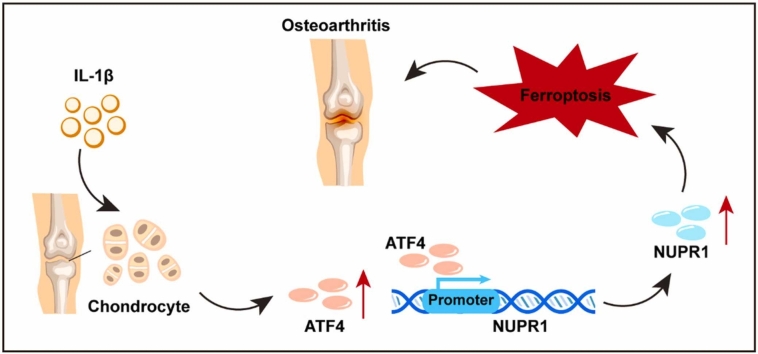
To describe the mechanism of action of ATF4 in OA. ATF4 expression is increased in IL-1β-treated chondrocytes and promotes ferroptosis through transcriptional activation of NUPR1, ultimately aggravating OA.

## 1 Introduction

Osteoarthritis (OA), a predominant degenerative joint disorder, is distinguished by the progressive deterioration of joint cartilage, remodeling of subchondral bone, and synovial inflammation [1], [2]. It represents the leading cause of disability worldwide, resulting in pain, reduced mobility, and a diminished quality of life [3]. In spite of considerable studies, the molecular interactions underlying OA etiology remain partially elucidated, hindering the advancement in developing effective therapeutic strategies.

Recent studies have highlighted ferroptosis, a manifestation of regulated cell apoptosis mediated by lipid peroxidation driven by iron, as a potential key mechanism in OA progression [4], [5]. This process may be particularly significant in chondrocytes, the primary cell type in cartilage [6]. Evidence supporting this hypothesis includes the finding that the synovial fluid of OA patients exhibited a notable elevation in iron levels, with serum ferritin concentrations illustrating a correlation with the extent of cartilage damage [7], [8]. Ferroptosis is initiated by an imbalance in oxidative homeostasis, giving rise to the aggregation of lipid-reactive oxygen species (ROS), which causes cell membrane rupture in sequence [9], [10]. Experimental studies have demonstrated that ferroptosis-specific inhibitors and iron chelators can reduce chondrocyte death and slow the progression of OA in animal models [11], [12]. These findings suggest that targeting the molecular mechanisms driving ferroptosis could offer novel therapeutic strategies to alleviate OA.

Among the molecular pathways implicated in ferroptosis, the role of activating transcription factor 4 (ATF4) has garnered increasing attention. ATF4 is a stress-responsive transcription factor activated during cellular stress, including endoplasmic reticulum (ER) stress [13]. During OA progression, activation mediated by ER stress of the protein kinase RNA-like endoplasmic reticulum kinase/eukaryotic initiation factor 2 alpha (PERK/eIF2α) signaling pathway leads to ATF4 upregulation, which promotes chondrocyte apoptosis and cartilage degradation [14]. Suppression of ATF4 expression has been shown to reduce cartilage damage and delay OA progression in animal models [15]. Furthermore, under inflammatory conditions, reduced selenoprotein M expression exacerbates ER stress by promoting protein misfolding and activating the PERK/p-eIF2α/ATF4 pathway, resulting in ferroptosis activation and increased cartilage damage [16]. Taken together, these outcomes indicate that ATF4 holds a crucial position in promoting ferroptosis as well as OA progression.

Nuclear protein 1 (NUPR1), a stress-induced protein, is another critical player in ferroptosis regulation. NUPR1 has been identified as a key suppressor of ferroptosis in cancer cells, contributing to tumor progression in liver and pancreatic cancers [17], [18]. Notably, our previous studies have revealed that silencing NUPR1 significantly reduces chondrocyte ferroptosis and alleviates OA progression [19]. Bioinformatics analysis using the JASPAR database [20] has further revealed that ATF4 directly binds to multiple sites in the NUPR1 promoter.

Based on literature research and database predictions, we hypothesize that ATF4 promotes chondrocyte ferroptosis by transcriptionally activating NUPR1, thereby accelerating OA progression. By elucidating the molecular mechanism through which the ATF4-NUPR1 axis promotes OA progression via ferroptosis, this research aims to provide a mechanistic foundation for novel therapeutic strategies targeting OA.

## 2 Methods

### 2.1 Cell culture and treatment

The human chondrocyte cell line C28/I2, obtained from the Cell Bank of the Chinese Academy of Sciences (Shanghai), was cultured in Dulbecco's Modified Eagle Medium (DMEM; Gibco, USA) supplemented with 10 % fetal bovine serum (FBS; Gibco) and 1 % penicillin-streptomycin (Gibco). Cells were maintained at 37 °C in a humidified incubator with 5 % CO₂. To simulate OA conditions, cells were treated with 10 ng/mL interleukin-1β (IL-1β; PeproTech, USA), following a previously described protocol [21]. Control cells were treated with an equivalent volume of PBS. All experiments were conducted in triplicate.

### 2.2 Cell transfection

For gene silencing or overexpression, transfection of cells was conducted via Lipofectamine 3000 (Invitrogen, USA) following the manufacturer’s protocol. Specifically, gene silencing was achieved by transfecting cells with 50 nM short hairpin RNA targeting ATF4 (sh-ATF4) or a non-targeting control (sh-NC), both synthesized by GenePharma Co., Ltd. (Suzhou, China). Gene overexpression was performed by transfecting cells with 2 µg/mL NUPR1 overexpression plasmid (oe-NUPR1), also synthesized by GenePharma Co., Ltd. (Suzhou). Following a 48-hour transfection period, cells underwent IL-1β treatment as described above.

### 2.3 Quantitative reverse transcription polymerase chain reaction（qRT-PCR）

Total RNA extraction was performed via TRIzol reagent (Invitrogen, USA), followed by reverse transcription into complementary DNA (cDNA) via the High-Capacity cDNA Reverse Transcription Kit (Applied Biosystems, USA). Quantitative reverse transcription polymerase chain reaction (qRT‑PCR) reactions were subsequently conducted on an ABI ViiA7 Sequence Detection System (Life Technologies, USA) via SYBR Green Master Mix (ABI). GAPDH served as an internal regulation for normalization. The 2⁻^ΔΔCt^ method was employed for measuring ATF4 as well as NUPR1 relative expression levels. Table 1 outlined the sequences of the primers synthesized by Tsingke Biotech Ltd.

**Table 1:** The primers used in the present study.

	Forward primer (5′−3′)	Reverse primer (5′−3′)
ATF4	CTTCACCTTCTTACAACCTCTTC	GTAGTCTGGCTTCCTATCTCC
NUPR1	ACCTTCCCACCAGCAACC	ACCTTTCCGGCCTCCACCTC
GAPDH	GAAGGTGAAGGTCGGAGTC	GAAGATGGTGATGGGATTTC

### 2.4 Western blot

Protein lysates were prepared using RIPA buffer (Thermo Fisher Scientific, USA) supplemented with protease and phosphatase inhibitors (Roche, Switzerland). The BCA Protein Assay Kit (Thermo Fisher Scientific, USA) was utilized for calculating protein concentrations. SDS-PAGE was employed for resolving equal quantities of protein, which were subsequently transferred to PVDF membranes sourced from Millipore, USA. Blocking of the membranes was performed via 5 % non-fat milk, followed by overnight incubation at 4 ℃ with major antibodies: anti-ATF4 at a dilution of 1:1000 (Abcam, ab216839, UK), anti-NUPR1 at a dilution of 1:1000 (Bioss, Catalog #: bs-7106R, China), anti-GPX4 (Glutathione Peroxidase 4) at a dilution of 1:1000 (Abcam, ab125066), anti-ACSL4 (Acyl-CoA Synthetase Long-Chain Family Member 4) at a dilution of 1:1000 (Abcam, ab155282), anti-SLC7A11 (Solute Carrier Family 7 Member 11) at a dilution of 1:1000 (Abcam, ab307601), and anti-GAPDH at a dilution of 1:5000 (Proteintech, 10494-1-AP). For 1-hour incubation at room temperature, secondary HRP-conjugated antibodies (1:5000; Cell Signaling Technology, USA) were utilized, followed by visualization of protein bands via an enhanced chemiluminescence detection system (Bio-Rad, USA).

### 2.5 Measurement of intracellular ROS, MDA, Fe^2+^, and GSH

Intracellular glutathione (GSH), malondialdehyde (MDA), ferrous iron (Fe²⁺), and ROS levels were measured to evaluate ferroptosis using commercially available assay kits (Elabscience, China). GSH levels were determined spectrophotometrically at 412 nm and ferrous ion levels at 593 nm by lysing cells and reacting the supernatant with specific detection reagents as instructed by the manufacturer’s protocol. MDA content was evaluated at 532 nm via a thiobarbituric acid reactive substances assay, involving the incubation of cell lysates with reaction buffer at 95 °C for 30 min, followed by cooling, centrifugation, and absorbance measurement. Intracellular Fe²⁺ levels were visualized using the Ferro Orange fluorescent probe (Dojindo Laboratories, Japan). Under conditions of darkness, cells were subjected to incubation with the probe at 37 °C for 30 min, and fluorescence was observed under a microscope. Lipid ROS were detected with the C11-BODIPY 581/591 fluorescence probe (Thermo Fisher Scientific, USA) by incubating cells with the probe at 37 °C for 30 min, and fluorescence signal shifts from red to green, indicative of lipid peroxidation, were analyzed microscopically.

### 2.6 Cell counting Kit-8 (CCK-8)

CCK8 assays were performed according to the manufacturer’s manual (Beyotime Biotechnology, Shanghai, China). Briefly, 2000 cells in 100 μL of culture medium were seeded into individual well of a 96-well plate and allowed to adhere for 24 h. After the desired incubation periods of 24, 48, as well as 72 h, 10 μL of sterile CCK-8 solution was administered to each well. Subsequently, the plate was subjected to an additional 2-hour incubation at 37 °C in a 5% CO₂ incubator to allow the formazan dye to develop. During this time, the cells metabolized the CCK-8 reagent, producing a color change proportional to cell viability. A microplate reader (Bio-Rad, USA) was employed for measuring the absorbance at 450 nm, serving as an indicator of cell viability.

### 2.7 Dual-luciferase reporter assay

A pGL3-basic luciferase reporter vector (Promega, USA) was utilized for cloning the promoter region of NUPR1. C28/I2 cells underwent co-transfection with the luciferase reporter plasmid, along with either sh-ATF4 or sh-NC using Lipofectamine 3000 (Invitrogen, USA). Renilla luciferase plasmid was induced in the co-transfection as an internal regulation. After an incubation time of 48 h, luciferase activity was quantified via the Dual-Luciferase Reporter Assay System (Promega, USA), with normalization to Renilla luciferase activity.

### 2.8 Chromatin immunoprecipitation followed by quantitative PCR (ChIP-qPCR)

The SimpleChIP Enzymatic Chromatin IP Kit (Cell Signaling Technology, USA) was adopted for chromatin Immunoprecipitation (ChIP). Cells were treated with 1% formaldehyde for 10 min to induce crosslinking, followed by chromatin fragmentation via sonication to an average size of 200–500 bp. Immunoprecipitation was carried out using an anti-ATF4 antibody (Abcam, ab85049) or normal IgG as a control. Protein A/G beads were utilized for capturing chromatin-antibody complexes, which were subsequently washed to eliminate non-specific bindings. Crosslinks were reversed by incubation at 65 °C, and DNA was purified. qPCR was performed with primers targeting ATF4-binding sites in the NUPR1 promoter, and enrichment was quantified using the comparative Ct method. The results revealed ATF4 binding to the NUPR1 promoter region.

### 2.9 Animal model and lentivirus injection

Male C57BL/6 mice (6–8 weeks old; Beijing Vital River Laboratory Animal Technology Co., Ltd., China) were housed under controlled conditions with a 12-h light/dark cycle at 22–24 °C and 40–60% humidity. These mice were randomly divided into four groups: sham, DMM, DMM + sh-NC, and DMM + sh-ATF4 (n = 6 per group). For ATF4 knockdown, lentiviruses carrying sh-ATF4 or sh-NC (GeneChem, Shanghai, China) were used for gene silencing. Two weeks prior to DMM surgery, the knee joint cavities of the mice received an injection of 20 μL of lentiviral suspension (1 × 10⁸ TU/mL). The lentiviral vectors were delivered into the synovial tissue, allowing efficient transfection into the knee joint structures. As previously outlined, the destabilization of the medial meniscus (DMM) model was generated via surgical destabilization of the medial meniscus to induce osteoarthritis [22]. Mice underwent anesthesia with isoflurane, followed by the creation of a medial parapatellar incision. The anterior attachment of the medial meniscus was cut, allowing it to destabilize. The surgical site was sutured, and mice were monitored during recovery. Knee joint tissues were harvested for histological and molecular analysis eight weeks post-surgery. This study was conducted under protocols approved by the Laboratory Animal Welfare and Ethics Committee of Nanjing University of Chinese Medicine (No.202302A016) and complied with the NIH Guide for the Care and Use of Laboratory Animals.

### 2.10 Histological analysis

Knee joint samples underwent 3-day fixation in 4% paraformaldehyde (PFA) for 3 days, 14-day decalcification in 14% EDTA solution at room temperature, as well as paraffin embedding. Using a microtome, the knee joint is cut from the sagittal plane into 3 µm thick slice with the patellar ligament as the centre. After deparaffinization and rehydration, sections underwent staining with hematoxylin and eosin (H&E) as well as safranin O-fast green for cartilage structure observation. Quantification of cartilage degeneration was performed via the Osteoarthritis Research Society International (OARSI) scoring system. Additionally, osteophyte and synovitis scores were used for further evaluation, as described in previous studies [23]. The ImageJ software was utilized for examining the extent of cartilage damage.

### 2.11 Statistical analysis

Triplicate experiments were conducted, with data reported as mean ± SD. GraphPad Prism (version 10.1.2) was utilized for statistical assessment. For two-group comparisons, a two-tailed unpaired Student’s t test was used. For multiple comparisons, one-way ANOVA followed by Tukey’s post hoc test was applied. P value < 0.05 was considered statistically significant.

## 3 Results

### 3.1 ATF4 was elevated in IL-1β-treated chondrocytes and facilitates ferroptosis

**Fig. 1:**
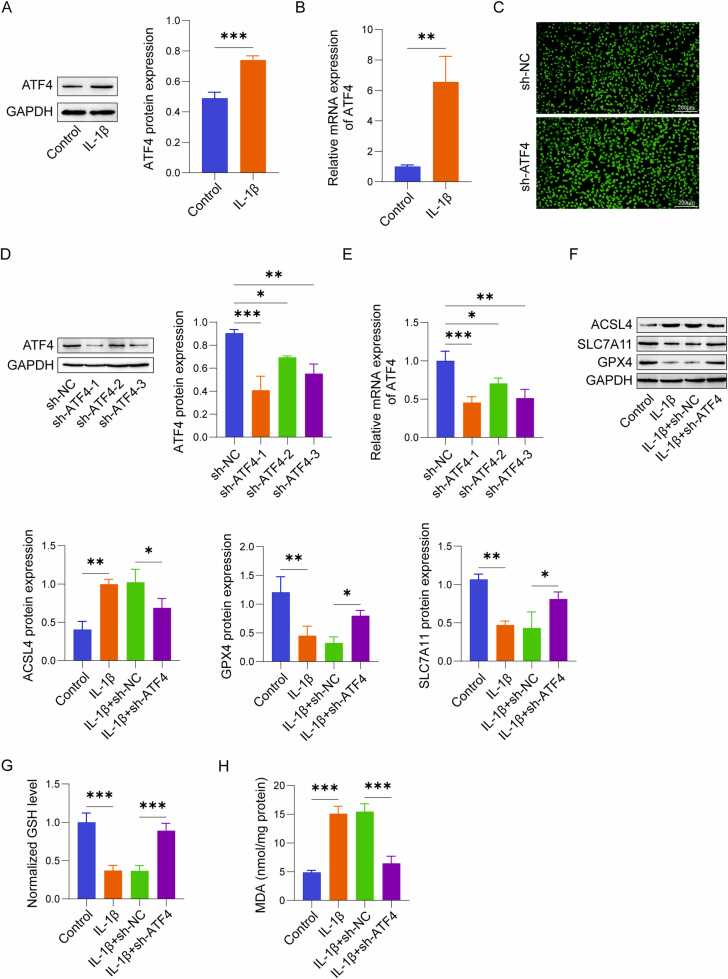

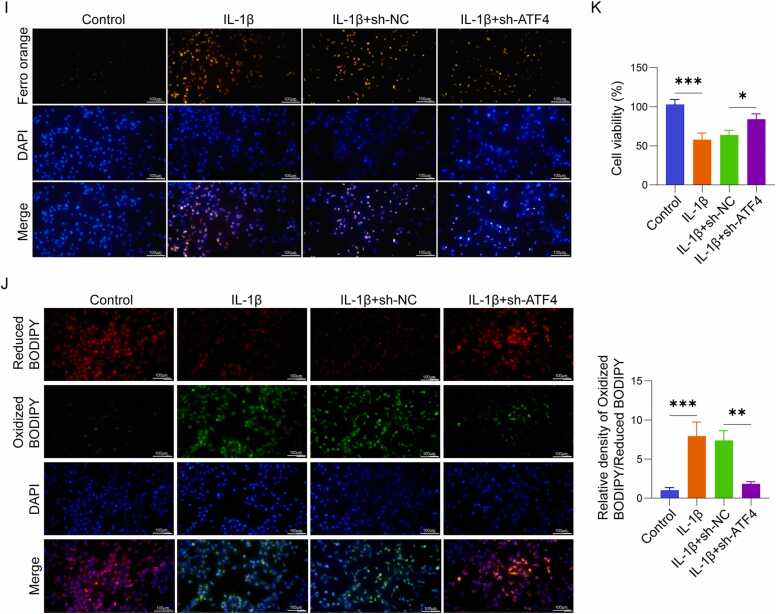
ATF4 was upregulated in IL-1β-treated chondrocytes and facilitates ferroptosis (n = 3). (A-B) ATF4 expression in human chondrocyte C28/I2 cells by qRT-PCR and western blot analysis. (C) Fluorescence microscopy to observe cell transfection efficiency. (D-E) qRT-PCR and western blot analysis of ATF4 expression levels after shRNA transfection. (F) Western blot analysis of ferroptosis-related proteins GPX4, ACSL4, and SLC7A11. (G-H) Measurement of GSH and MDA levels using specific assay kits. (I) Ferro Orange fluorescent probe detection of Fe²⁺ levels. (J) C11-BODIPY 581/591 fluorescent probe detection of lipid ROS levels. (K) CCK-8 assay for determining cell viability. *P < 0.05, **P < 0.01, and ***P < 0.001.

To examine the impact of ATF4 in OA, we evaluated its expression and related cellular changes in IL-1β-treated chondrocytes. IL-1β stimulation significantly increased ATF4 expression in C28/I2 chondrocytes, as shown by qRT-PCR as well as western blot. (Fig. 1A, B). After 48 h of cell infection, infection efficiency was observed using fluorescence microscopy, with over 90% of cells showing fluorescence (Fig. 1C). sh-ATF4 effectively downregulated ATF4 expression in C28/I2 cells, with the first sh-RNA demonstrating the highest knockdown efficiency. The sh-ATF4–1 was selected for subsequent studies, as validated via qRT-PCR as well as western blot analysis (Fig. 1D, E). To investigate the impact of ATF4 on ferroptosis in IL-1β-stimulated chondrocytes, we observed the expression of ferroptosis-related proteins in C28/I2 cells. Following IL-1β stimulation, expression of ferroptosis-associated proteins GPX4 and SLC7A11 was significantly decreased, while ACSL4 expression was increased (Fig. 1F). In addition, the level of GSH was significantly reduced (Fig. 1G), while MDA content was markedly increased (Fig. 1H). In addition, IL-1β treatment caused a notable elevation in Fe²⁺ fluorescence intensity in the cells (Fig. 1I). In C11-BODIPY 581/591 fluorescence staining, IL-1β treatment increased the green fluorescence in chondrocytes, indicating lipid peroxidation (Fig. 1J). Furthermore, cell viability was significantly reduced due to IL-1β stimulation (Fig. 1K). However, after ATF4 knockdown, these indicators were significantly reversed, showing a downregulation of ferroptosis-related genes, reduced Fe²⁺ fluorescence intensity, a more reduced ROS profile, and increased cell viability (Fig. 1F-K). These results suggest that IL-1β induces ATF4 expression in chondrocytes, which is linked to an elevation in ferroptosis. Knockdown of ATF4 significantly inhibits ferroptosis and improves chondrocyte viability, underscoring ATF4 as a potential modulator of ferroptosis in cartilage cells under inflammatory conditions.

### 3.2 ATF4 knockdown alleviated ferroptosis and OA progression in DMM mice

**Fig. 2:**
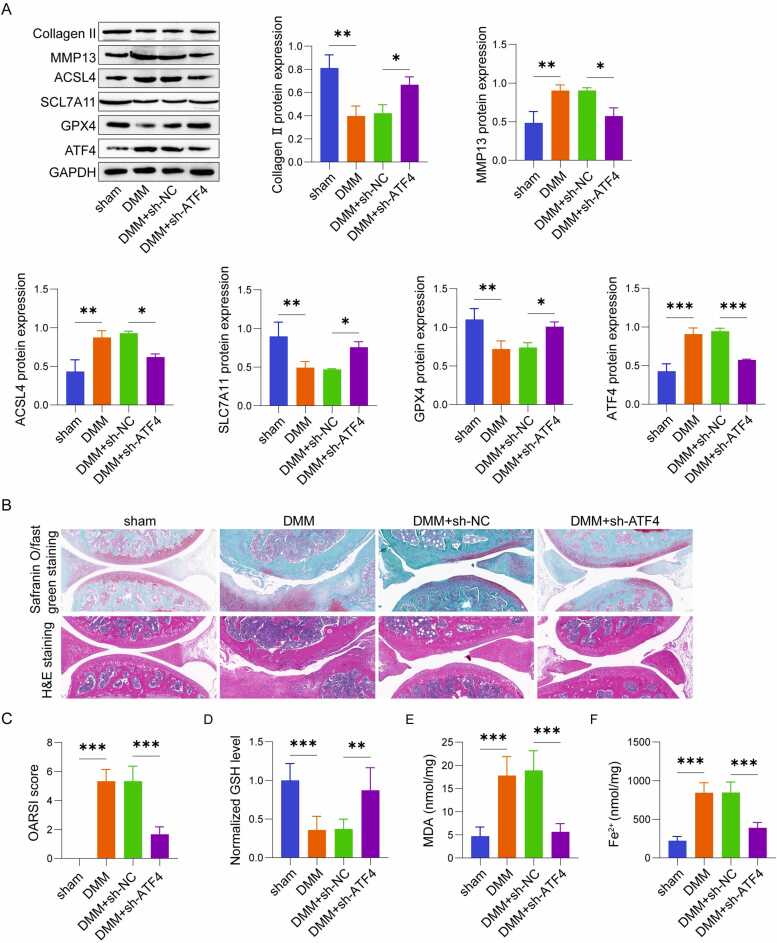
ATF4 knockdown alleviated ferroptosis and OA progression in DMM mice (n = 6). (A) Western blot analysis of ATF4, GPX4, ACSL4, SLC7A11, MMP13, and Collagen II expression in mouse cartilage tissues. (B) Histopathological analysis of mouse cartilage using H&E staining and Safranin O-Fast Green staining to assess pathological changes. The right side of the image represents the anterior aspect of the knee joint. Representative images are shown; similar pathological changes were observed in at least 5 out of 6 mice in each group. (C) OARSI scoring to evaluate OA progression. (D-F) Measurement of GSH, MDA, and Fe²⁺ levels in cartilage tissues using specific assay kits. *P < 0.05, **P < 0.01, and ***P < 0.001.

To further assess the role of ATF4 in OA, we examined its functions in a DMM mouse model. Compared to sham-operated controls, ATF4 expression was significantly elevated in knee cartilage from DMM mice, leading to ferroptosis and an increased expression of matrix metalloproteinase MMP13, and a marked decrease in Collagen II expression (Fig. 2A). However, in mice pretreated with intra-articular injection of sh-ATF4 lentivirus two weeks before DMM surgery, these pathological changes were reversed. Specifically, sh-ATF4 treatment restored GPX4 and SLC7A11 expression, reduced ACSL4 and MMP13 levels, and increased Collagen II expression (Fig. 2A). Histological analysis further confirmed the protective effects of ATF4 knockdown. In sham-operated mice, cartilage morphology was intact, with orderly cell arrangement and an OARSI score of 0. In contrast, DMM mice displayed evident cartilage damage, including reduced cartilage thickness, fibrotic changes on the cartilage surface, and partial cartilage defects, resulting in significantly increased OARSI scores (Fig. 2B, C). In the DMM + sh-ATF4 group, cartilage integrity was markedly improved, with increased cartilage thickness and reduced fibrosis, leading to significantly decreased OARSI scores compared to the DMM + sh-NC group (Fig. 2B, C). Biochemical analyses revealed additional evidence of ferroptosis in the cartilage of DMM mice. GSH levels were significantly reduced, while MDA content and Fe²⁺ levels were markedly elevated in DMM mice compared to sham controls (Fig. 2D-F). However, in mice treated with sh-ATF4, these ferroptosis markers were significantly reversed, indicating ferroptosis inhibition in the knee cartilage of mice (Fig. 2D-F). Generally, these outcomes demonstrate that ATF4 is upregulated in the knee cartilage of DMM mice and exerts a fundamental effect in ferroptosis as well as OA progression. Knockdown of ATF4 effectively suppresses ferroptosis, improves cartilage integrity, and mitigates OA progression.

### 3.3 ATF4 transcriptionally activated NUPR1

**Fig. 3:**
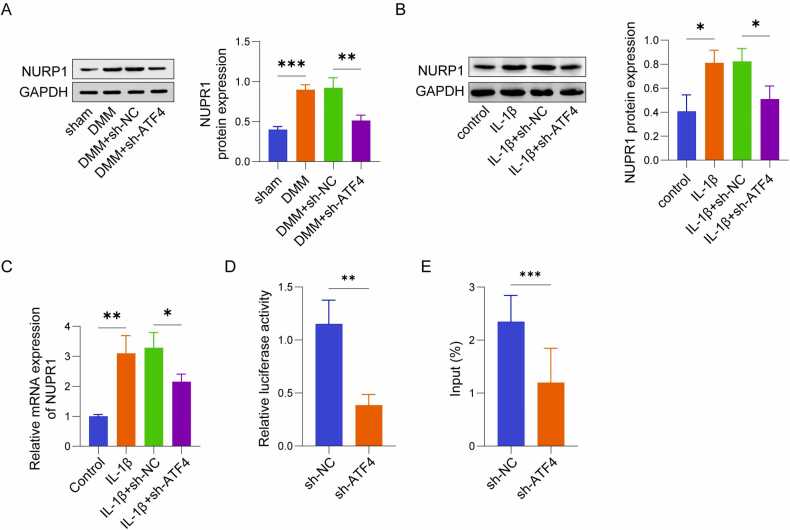
ATF4 transcriptionally activated NUPR1 (n = 3). (A)Western blot analysis of NUPR1 expression in articular cartilage. (B-C) qRT-PCR and western blot analysis of NUPR1 expression levels in human chondrocyte C28/I2 cells. (D) Dual-luciferase assay demonstrating the interaction between ATF4 and the NUPR1 promoter. (E) ChIP-qPCR analysis showing the enrichment of ATF4 at the NUPR1 promoter region. *P < 0.05, **P < 0.01, and ***P < 0.001.

Our previous study demonstrated that knockdown of NUPR1 delays OA pathological progression by inhibiting chondrocyte ferroptosis [19]. Combining this with predictions from the JASPAR database, we further explored the mechanism by which ATF4 regulates ferroptosis in OA. To validate the role of ATF4 in regulating NUPR1 expression in vivo, we first examined NUPR1 protein levels in cartilage tissues. Western blot analysis showed that NUPR1 expression was upregulated in the cartilage of DMM mice compared to sham controls, and its expression decreased following ATF4 knockdown (Fig. 3A). Consistent with the in vivo findings, IL-1β treatment significantly elevated NUPR1 mRNA and protein levels in C28/I2 chondrocytes in vitro However, when ATF4 was knocked down, NUPR1 expression was markedly reduced (Fig. 3B, C). To further confirm the transcriptional regulation of NUPR1 by ATF4, a luciferase reporter plasmid incorporating the NUPR1 promoter sequence was generated and transfected into chondrocytes. Cells were subsequently underwent co-transfection with either sh-NC or sh-ATF4. The luciferase activity assay revealed that knockdown of ATF4 significantly decreased luciferase activity, indicating that ATF4 transcriptionally activated NUPR1 by directly interacting with its promoter region (Fig. 3D). Additionally, ChIP-qPCR analysis revealed an enrichment of ATF4 at the NUPR1 promoter region in IL-1β-stimulated chondrocytes. This enrichment was significantly reduced following ATF4 knockdown, providing further evidence of ATF4's direct binding to the NUPR1 promoter (Fig. 3E). These findings suggest that in IL-1β-stimulated human chondrocytes, ATF4 transcriptionally activates NUPR1 by binding to its promoter, thereby contributing to its upregulation.

### 3.4 ATF4 promoted ferroptosis in IL-1β-induced chondrocytes via NUPR1

**Fig. 4:**
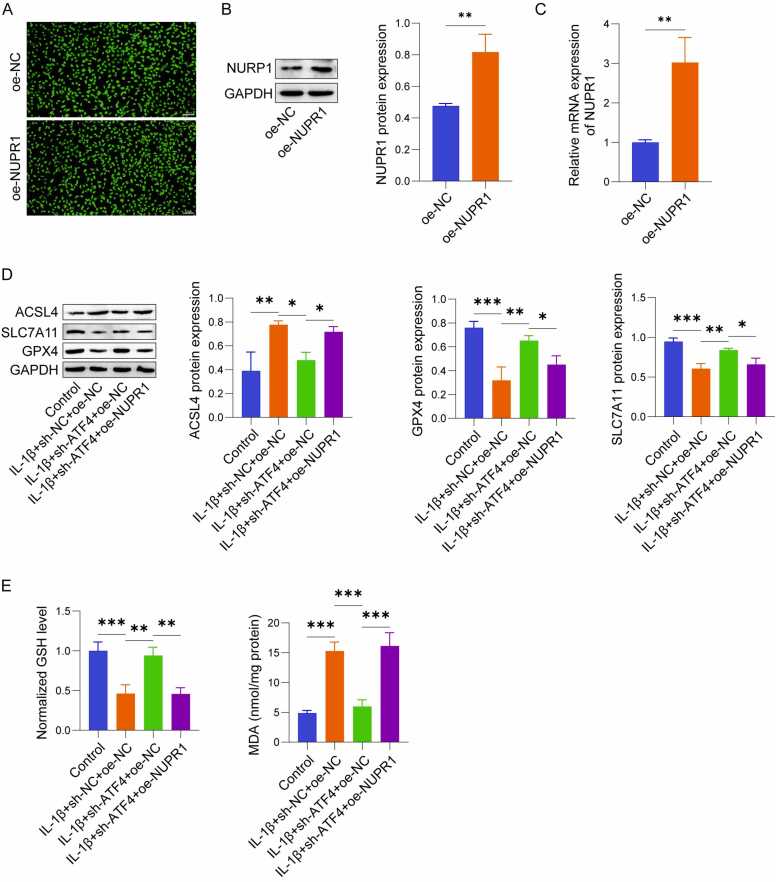

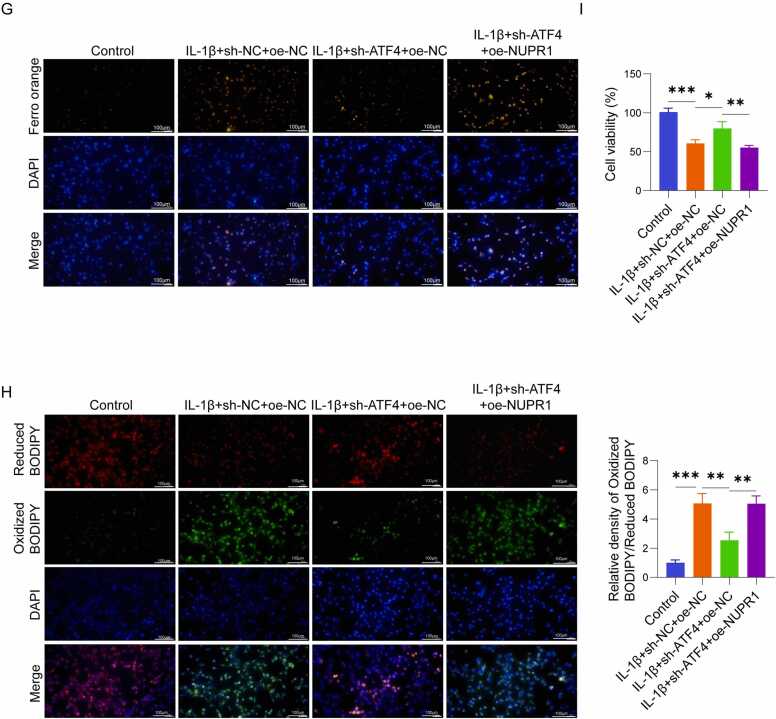
ATF4 promoted ferroptosis in IL-1β-induced chondrocytes via NUPR1 (n = 3). (A-C) Fluorescence microscopy at 48 h post-transfection and western blot/ qRT-PCR analysis of NUPR1 expression levels. (D) Western blot analysis of ferroptosis-related proteins GPX4, ACSL4, and SLC7A11. (E-F) Measurement of GSH and MDA levels in chondrocytes using assay kits. (G) Detection of Fe²⁺ levels using Ferro Orange fluorescent probes. (H) Lipid ROS levels detected by C11-BODIPY 581/591 fluorescent probes, showing changes in red and green fluorescence. (I) CCK-8 assay for evaluating cell viability. *P < 0.05, **P < 0.01, and ***P < 0.001.

Finally, we investigated whether ATF4 influences ferroptosis in IL-1β-stimulated chondrocytes through NUPR1. After 48 h of infection, fluorescence microscopy revealed that over 90% of cells exhibited fluorescence, confirming successful transfection (Fig. 4A). oe-NUPR1 significantly increased NUPR1 expression in C28/I2 cells, as verified via qRT-PCR as well as western blot analysis (Fig. 4B, C). In C28/I2 cells stimulated by IL-1β, ferroptosis was notably increased, accompanied by a significant reduction in cell viability. Knockdown of ATF4 effectively suppressed ferroptosis and restored cell viability, as indicated by changes in ferroptosis-related markers and functional assays. However, when NUPR1 was overexpressed simultaneously in ATF4 knockdown cells, the protective effects of ATF4 knockdown were reversed, leading to increased ferroptosis. This outcome was validated by the downregulation of GPX4 and SLC7A11, upregulation of ACSL4 (Fig. 4D), reduced GSH levels (Fig. 4E), elevated MDA levels (Fig. 4F), and increased Fe²⁺ fluorescence intensity (Fig. 4G). Additionally, NUPR1 overexpression restored lipid peroxidation, as shown by the increased green fluorescence and decreased red fluorescence in C11-BODIPY 581/591 staining (Fig. 4H), and significantly reduced cell viability (Fig. 4I). These findings indicate that ATF4 promotes ferroptosis in chondrocytes triggered by IL-1β through upregulating NUPR1. Knockdown of ATF4 suppresses ferroptosis and restores cell viability, but these effects are reversed when NUPR1 is simultaneously overexpressed, highlighting its critical role in this regulatory pathway.

## 4 Discussion

The primary features of OA, a progressive degenerative joint disorder, encompass cartilage degeneration, synovial inflammation, and subchondral bone remodeling [1]. Recent research has highlighted the significance of ferroptosis in the etiology of OA. Ferroptosis contributes to cartilage degradation by promoting chondrocyte death under inflammatory conditions [4], [5]. In this study, we found that ATF4 regulated ferroptosis and promoted OA progression by transcriptionally activating NUPR1.

ATF4 has been identified as an important modulator of cellular stress responses, including oxidative stress, inflammation, and cell survival [24]. Our findings showed that IL-1β treatment significantly upregulated ATF4 expression in chondrocytes, leading to ferroptosis increased, including altered levels of key proteins involved in oxidative stress and lipid peroxidation. In vivo, ATF4 was notably elevated in the knee cartilage of DMM mice as opposed to sham-operated controls, and its expression correlated with ferroptosis-related changes, alongside decreased Collagen II expression. These outcomes align with prior research that inflammation as well as oxidative stress exert fundamental effects in driving ferroptosis in OA [25], [26]. To substantiate ferroptosis as the primary mode of cell death in this context, we evaluated multiple ferroptosis indicators, including GPX4, SLC7A11, ACSL4, GSH, MDA, lipid ROS, and Fe²⁺, which are broadly recognized as hallmarks of ferroptosis due to their critical roles in redox homeostasis, lipid peroxidation, and iron dependency [27], [28], [29]. Our study provides direct evidence that ATF4 is a key player in this process. Knockdown of ATF4 reversed the IL-1β-induced ferroptotic markers, reducing Fe²⁺ accumulation, and mitigating lipid peroxidation, as evidenced by the reduction in green fluorescence in C11-BODIPY staining. This was further supported by the increased cell viability observed following ATF4 knockdown. Knockdown of ATF4 in DMM mice also reversed these changes, further confirming the reduction in ferroptotic activity in knee cartilage. In addition, histological analysis showed that ATF4 knockdown significantly improved cartilage morphology and reduced OARSI scores. Biochemical analyses revealed reduced GSH, as well as elevated MDA and Fe²⁺ levels in DMM mice, indicative of ferroptosis. These outcomes underscore the crucial function of ATF4 in ferroptosis modulation in OA, indicating its possible application as a therapeutic target to alleviate cartilage damage by modulating ferroptosis in OA.

ATF4 functions as a transcriptional activator, modulating various downstream proteins involved in cellular stress responses. For instance, ATF4 has been shown to regulate the expression of GDF15, which inhibits inflammation triggered by LPS and MUC5AC expression in human nasal epithelial cells [30]. Other study has demonstrated that ATF4 regulates NADPH production by controlling the expression of key enzymes in the pentose phosphate pathway and mitochondrial serine/glycine/folate metabolism, thereby maintaining redox balance and mitigating oxidative stress under hemodynamic stress [31]. In our study, we identified NUPR1 as one of the key downstream effector of ATF4 in chondrocytes. Knockdown of ATF4 significantly reduced NUPR1 expression, consistent with previous findings that ATF4 can transcriptionally activate NUPR1 under oxidative stress conditions [32]. Interestingly, while NUPR1 has been reported to suppress ferroptosis in various cancers by maintaining mitochondrial integrity and iron homeostasis, our previous research demonstrated that NUPR1 knockdown inhibits chondrocyte ferroptosis and delays OA progression [19]. In this study, we further show that ATF4 promotes ferroptosis by activating NUPR1 in IL-1β-stimulated chondrocytes. These results, together with recent evidence that NUPR1 enhances ferroptosis and accelerates osteoblast aging [33], indicate that NUPR1 may exert dual, context-dependent roles in ferroptosis regulation. In tumor environments, NUPR1 likely suppresses ferroptosis to support cell survival, whereas in OA—characterized by chronic inflammation and oxidative stress—it may facilitate ferroptosis and cartilage degradation. This functional plasticity of NUPR1 in response to different cellular stressors suggests an adaptive mechanism that contributes to disease progression in distinct pathological settings and warrants further investigation. Moreover, NUPR1 is a transcriptional regulator that can modulate ferroptosis by simultaneously influencing both pro-ferroptotic and anti-ferroptotic genes through distinct signaling pathways. For example, Huang et al. demonstrated that NUPR1 modulates ferroptosis through its involvement in multiple pathways, including iron metabolism, ROS homeostasis, and the GSH/GPX4 antioxidant axis [32]. In addition, Tan et al. revealed that NUPR1 suppresses ferroptosis in triple-negative breast cancer by downregulating ACSL4 via the NUPR1–LCN2 axis [34]. These findings suggest that NUPR1 may coordinate opposing ferroptosis-related gene networks through multiple regulatory routes.

The relationship between ATF4 and NUPR1 in OA is particularly important, as it suggests that ATF4, by activating NUPR1, could modulate the interplay between cell viability and cell mortality under inflammatory conditions. NUPR1's role in protecting cells from oxidative stress and ferroptosis might explain why ATF4 is essential for chondrocyte survival and cartilage integrity in OA [35], [36]. NUPR1′s involvement in ferroptosis regulation has been previously suggested by Liu et al. [37], who has linked NUPR1 to the cellular response to oxidative stress and iron accumulation. Our findings build on this work by showing that ATF4 activates NUPR1 transcriptionally, which in turn enhances iron accumulation and lipid peroxidation, key processes in ferroptosis [38]. This mechanism highlights the ATF4-NUPR1 pathway as a potential therapeutic target for controlling ferroptosis in OA, suggesting that modulating this pathway could help preserve cartilage integrity and slow disease progression.

While our observations shed light on the function of ATF4 in ferroptosis as well as OA, several limitations should be considered. First, the study primarily relies on in vitro and animal models, which may not fully recapitulate the complexity of human OA. Future studies should include clinical samples from OA patients to validate the expression and role of ATF4 and NUPR1 in human cartilage. Second, although we identified NUPR1 as a key downstream target of ATF4, ATF4 is known to regulate a broad range of genes involved in cellular stress responses. Thus, it is likely that NUPR1 represents one of several important mediators, rather than the sole effector of ATF4′s actions in osteoarthritis. Additional ATF4-regulated targets, such as those involved in iron metabolism, redox homeostasis, or mitochondrial function, may also contribute to ferroptosis and OA progression. Future studies, such as ChIP-seq are needed to explore additional ATF4-regulated genes involved in OA-related ferroptosis. Third, the long-term effects of ATF4 inhibition on cartilage repair and joint function remain to be assessed, as chronic inhibition could potentially lead to unwanted side effects, such as impaired tissue repair or enhanced susceptibility to infection [39]. Long-term in vivo studies are essential for assessing the security and efficacy of targeting ATF4 and NUPR1 in OA therapy. Moreover, it is important to consider that OA is a multifactorial disease influenced by biochemical, mechanical, and immunological factors. Recent studies suggest that mechanical stress induces ferroptosis in chondrocytes via Piezo1-mediated Ca²⁺ influx and GPX4 suppression [40], and immune responses have also been shown to interact with ferroptosis-related pathways and contribute to OA progression [41]. Future studies should integrate these factors to provide a more comprehensive understanding of ferroptosis regulation in OA.

## 5 Conclusions

This research reveals that ATF4 exerts a pivotal effect in promoting ferroptosis in chondrocytes and driving OA progression, at least in part, by transcriptionally activating NUPR1. The ATF4-NUPR1 axis plays an important role in regulating ferroptosis, contributing to cartilage degeneration and disease progression in OA. Our findings offer novel perspectives on the molecular mechanisms underpinning OA and underscore ATF4 and NUPR1 as potential therapeutic targets for managing OA. Targeting this pathway could offer new strategies for preserving cartilage integrity and slowing the progression of OA, opening avenues for potential therapeutic developments in the treatment of this debilitating disease. Additional research is required to verify these findings in human OA and investigate the potential clinical utility of modulating the ATF4-NUPR1 pathway in OA treatment.

## CRediT authorship contribution statement

**Peimin Wang:** Writing – review & editing, Supervision. **Liang Ding:** Writing – review & editing, Supervision. **Enrui Hu:** Writing – review & editing, Visualization, Project administration. **Deren Liu:** Writing – review & editing, Visualization, Project administration, Data curation. **Yibao Wei:** Writing – review & editing, Formal analysis. **Lishi Jie:** Writing – review & editing, Formal analysis, Conceptualization. **Taiyang Liao:** Writing – review & editing, Writing – original draft, Conceptualization. **Chen Kuang:** Writing – review & editing, Writing – original draft, Conceptualization.

## Ethics approval and consent to participate

This study was conducted under protocols approved by the Laboratory Animal Welfare and Ethics Committee of Nanjing University of Chinese Medicine (No.202302A016) and complied with the NIH Guide for the Care and Use of Laboratory Animals.

## Author contributions

Peimin Wang and Liang Ding guaranteed the integrity of the entire study. Chen Kuang and Taiyang Liao designed the study and intellectual content. Lishi Jie defined the literature research. Deren Liu and Enrui Hu performed experiment. Deren Liu collected the data. Yibao Wei and Lishi Jie analyzed the data. Chen Kuang and Taiyang Liao wrote the main manuscript and prepared figures. All authors reviewed the manuscript.

## Funding

This work was supported by the Jiangsu Provincial Medical Key Discipline (Laboratory) Cultivation Unit (Grant No. JSDW202252), the Clinical Medical Innovation Center for Knee Osteoarthritis in Jiangsu Province Hospital of Chinese Medicine (Grant No. Y2023zx05), and the Nanjing University of Chinese Medicine Clinical Research Institute for Knee Osteoarthritis (Grant No. LCZBYJYZZ2024-003).

## Consent for publication

Not Applicable.

## Declaration of Generative AI and AI-assisted technologies in the writing process

During the preparation of this work the authors did not use any AI-assisted technology.

## Declaration of Competing Interest

The authors declare that they have no known competing financial interests or personal relationships that could have appeared to influence the work reported in this paper.

## Data Availability

The datasets used or analyzed during the current study are available from the corresponding author on reasonable request.
